# Are We Doing Enough to Stem the Tide of Acquired MDR-TB in Countries with High TB Burden? Results of a Mixed Method Study in Chongqing, China

**DOI:** 10.1371/journal.pone.0088330

**Published:** 2014-02-05

**Authors:** Ying Li, John Ehiri, Eyal Oren, Daiyu Hu, Xingneng Luo, Ying Liu, Daikun Li, Qingya Wang

**Affiliations:** 1 Department of Social Medicine and Health Service Management, Third Military Medical University, Chongqing, China; 2 Division of Health Promotion Sciences, Mel & Enid Zuckerman College of Public Health University of Arizona, Tucson, Arizona, United States of America; 3 Division of Epidemiology and Biostatistics, Mel & Enid Zuckerman College of Public Health University of Arizona, Tucson, Arizona, United States of America; 4 Chongqing Institute of TB Prevention and Treatment, Jiulongpo District, Chongqing, China; 5 Department of TB control, Center of Disease Control in Shapingba District, Chongqing, China; 6 Department of Laboratory Medicine, University-Town Hospital of Chongqing Medical University, Chongqing, China; McGill University, Canada

## Abstract

Multi-drug resistant tuberculosis (MDR-TB) represents a threat to health and development in countries with high TB burden. China’s MDR-TB prevalence rate of 6.8% is the highest in the world. Interventions to remove barriers against effective TB control, and prevention of MDR-TB are urgently needed in the country. This paper reports a cross-sectional questionnaire survey of 513 pulmonary TB (PTB) patients, and qualitative interviews of 10 healthcare workers (HCWs), and 15 PTB patients. The objective was to assess barriers against effective control of PTB and prevention of MDR-TB by elucidating the perspectives of patients and healthcare providers. Results showed that more than half of the patients experienced patient delay of over 12.5 days. A similar proportion also experienced detection delay of over 30 days, and delay in initiating treatment of over 31 days. Consulting a non-TB health facility ≥3 times before seeking care at TB dispensary was a risk factor for both detection delay [AOR (95% CI): 1.89(1.07, 3.34) and delay in initiating treatment[AOR (95% CI): 1.88 (1.06, 3.36). Results revealed poor implementation of Directly Observed Therapy (DOT), whereby treatment of 34.3% patients was never monitored by HCWs. Only 31.8% patients had ever accessed TB health education before their TB diagnosis. Qualitative data consistently disclosed long patient delay, and indicated that patient’s poor TB knowledge and socioeconomic barriers were primary reasons for patient delay. Seeking care and being treated at a non-TB hospital was an important reason for detection delay. Patient’s long work hours and low income increased risk for treatment non-adherence. Evidence-based measures to improve TB health seeking behavior, reduce patient and detection delays, improve the quality of DOT, address financial and system barriers, and increase access to TB health promotion are urgently needed to address the burgeoning prevalence of MDR-TB in China.

## Introduction

While much progress has been made in global efforts to control tuberculosis (TB), significant challenges remain, particularly with regard to stemming the increasing prevalence of multidrug-resistant TB (MDR-TB) [1]. Globally, 3.7% of new cases and 20% of previously treated cases are estimated to have MDR-TB. There were an estimated 310,000 MDR-TB cases among notified TB patients with pulmonary TB (PTB) in 2011. Unfortunately, the global prevalence of MDR-TB is driven by cases in a few high-burden resource-poor countries, with India, China and the Russian Federation contributing about 60% of the cases [1], [2].

Currently, MDR-TB is more likely to be acquired than primary MDR-TB, with a prevalence of primary MDR of 1.4% compared to acquired resistance of 13% among individuals previously treated with TB medications [3]. Given that there is no effective vaccine to prevent MDR-TB and the challenges in diagnosing and treating the condition [1], [4]–[6], it is critically important to identify the factors that contribute to acquired MDR-TB, so that corresponding preventive measures can be implemented. Numerous studies have explored social and behavioral factors that are associated with the development of MDR-TB from general TB (drug-susceptible TB). These include the presence of HIV infection [7], history of imprisonment [7], disability sufficient to prevent work [7], alcohol abuse [7]–[8], smoking [7], poverty [9], lack of health insurance [10], and previous history of TB treatment [7]–[16]. Explanations for previous treatment as a risk factor for MDR-TB vary and may include incomplete treatment [9], [16]–[17], inappropriate and irregular intake of prescribed medications by patients [17], inadequate or irregular supply of drugs [6], [12]–[14], [18], and lack of treatment supervision (poor quality DOTS) [9], [19], patient delay, diagnostic delay, detection delay, and delay in initiating TB treatment [10], [11].

China carries the greatest burden of MDR-TB worldwide [1]. Although the Chinese government has initiatives for MDR-TB Control [20], the prevalence of MDR-TB continues to increase. Resistance to first-line drugs among newly diagnosed TB patients rose from 34.2% in 2007–2008 [16] to 36.9% in 2010 [21]. In China, the rate of MDR-TB among retreated TB cases (25.6%) is much higher than that of newly diagnosed TB patients (5.7%) [16]. Therefore, general TB (drug-susceptible TB) control is critically important for MDR-TB prevention and control in China. However, in spite of concerted international and national efforts to address TB in China, results of the 5th National TB Survey in 2010 indicated that only 47% of patients with TB symptoms sought healthcare in a timely fashion and only 59% adhered to prescribed treatment [21]. In order to provide evidence for preventing general TB from progressing to MDR-TB, this study assessed barriers in current TB control processes (including TB healthcare seeking, treatment, and access to TB health promotion) in Chongqing Municipality, which has one of the highest burdens of TB and MDR-TB in China. The study examined barriers from the perspectives of both TB patients and TB service providers, using a mixed method research approach.

## Materials and Methods

### Study Setting

We conducted a cross-sectional study in Chongqing Municipality from May 2012 to May 2013. Chongqing is one of four municipalities directly under the Central Government and located in the upper reaches of the Yangtze River, linking central and western China. Chongqing Municipality has three million urban residents, with the highest prevalence of TB in the country [21], [22]. The challenges of TB control in Chongqing include increasing prevalence of MDR-TB, TB among migrant populations and comorbidity of the human immunodeficiency virus (HIV) and TB [23]. Rates of DR-TB and MDR-TB were 14.9% and 4.6% respectively in 2005 [23]. The study sites were selected from six urban districts based on the following considerations: representation of relatively developed and less developed areas of Chongqing Municipality (based on average household income), willingness to participate in the study, and the capacity to organize the logistics for implementing the study. The three districts selected as study sites were YZ in central Chongqing, and JLP, and SPB in suburban Chongqing. There is one TB dispensary in each district.

### Definition of Terms

**Figure 1:**
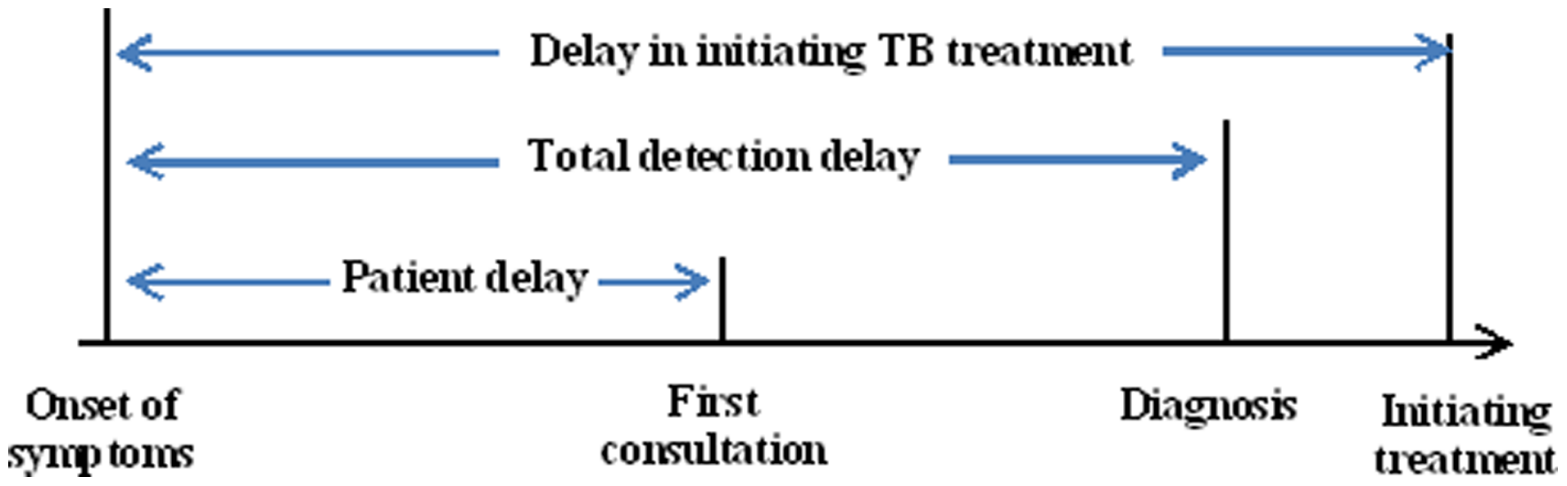
Definitions of and relations between the different types of delay among tuberculosis patients. This figure indicates the operational definitions for patient delay, total detection delay and delay in initiating treatment. It also presented the relations of the three types of delays.

Patient delay was defined as the time interval between onset of TB symptoms and the patient’s first presentation to a TB health facility [27]. The detection delay was defined as the time between onset of symptoms and actual diagnosis [28]. Delay in initiating TB treatment referred to the time between onset of symptoms and presentation for treatment during the most recent TB episode [14] (Figure 1). Interrupted treatment is defined as the discontinuation of medication for 2–8 consecutive weeks, before restarting treatment [29]. The cutoff point(s) for longer delays was defined according to the median values of each type of delay in this study.

### Data Collection

Qualitative and quantitative data inform each other and produce insight and understanding in a way that cannot be duplicated by either approach alone [24]. Increasingly researchers use a mixed-method strategy, as they combine qualitative and quantitative data to answer questions of interest [24]. This study combined quantitative study and qualitative study to collect data related to behaviors on TB care seeking, treatment and associated factors.

#### Questionnaire survey

All adult PTB patients who met the following criteria were targeted for recruitment in the 3 selected districts: (1) registered at TB dispensaries and were diagnosed as having PTB according to national TB program (NTP) criteria [25]; (2) newly diagnosed and retreatment PTB patients diagnosed in the past 6 months and who received anti-TB drugs treatment for at least two months; (3) aged 15 years and older. The expected prevalence of detection delay was 50% using median of detection delay as the cut-off point [21]. With a type I error rate of 5% and an expected relative error rate of 10–15%, using sample size estimation methods for cross-sectional studies [26], the estimated sample size for the study was approximately 400 patients. Logistics for recruitment was facilitated by TB Dispensaries in the selected study districts. During recruitment, potential participants were approached and provided with detailed explanation about the study and its objectives. They were then asked if they would be interested in volunteering to participate. Those who expressed interest were asked to read the informed consent form, and were assured of confidentiality. They were then asked to sign the informed consent form as a confirmation of their voluntary participation in the study. Similarly, the informed consent was thoroughly explained to potential participants who could not read or write. Those who agreed to participate were asked to give their thumb print as a confirmation of their voluntary participation.

A structured questionnaire survey was conducted in clinic rooms at the district TB dispensaries. Data collected included socio-demographic information, clinical features of TB (newly diagnosed/retreatment TB, smear positive/negative TB, typical TB symptoms including chronic cough, haemoptysis, fever, night sweats, weakness and weight loss), patient behaviors related to healthcare seeking and treatment (interval between onset of symptoms and seeking care in health facility for the first time, date of care seeking for the first time, first health facility visited, date of TB diagnosis, interval between diagnosis of TB and starting treatment, behaviors related to taking anti-TB drugs), health worker behaviors related to managing and educating TB patients, TB knowledge among TB patients, and patients’ access to TB health promotion.

#### Qualitative research

Ten healthcare workers (HCWs) who had been TB HCWs in TB dispensaries for more than ten years were purposively selected for key informant interview. Fifteen newly registered TB patients who experienced longer delays (more than 31 days) in initiating treatment were purposively selected for the in-depth interviews. Topic guides were used for interviews. Topic guides for key informant interviews included information about patients’ behaviors related to healthcare seeking and compliance with TB treatment, HCWs behaviors related to treatment of TB patients, and health education for TB patients. Topic guides for in-depth interviews elicited information about patients’ health seeking behaviors, adherence to TB treatment, and the patients’ perspectives about factors affecting their behaviors and access to TB diagnosis, treatment and health education. All interviews were recorded with respondents’ consent. Each interview lasted about 40–60 minutes.

### Data Analysis

#### Quantitative analysis

Data were entered using Epi Data 3.1. The data were analyzed using the Statistical Package for Social Science (SPSS 18.0) (IBM Corporation, Armonk, NY, USA). A two-tailed probability level of p<0.05 was chosen as the level of statistical significance. Missing data were excluded from analysis. Descriptive statistics were used to present study participants’ characteristics, adherence to treatment, healthcare workers’ behaviors related to DOT, health promotion, and patient-reported access to TB health promotion before infection with TB. Patient delay, total detection delay, and delay in initiating treatment were also summarized using mean (standard deviation, SD), median (interquartile range, IQR), and percentage of patients with longer delay (delay ≥median). Demographic and clinical characteristics of the patients with delay were compared with those of patients without delay. The Chi-square test was used to compare characteristics of the two groups. Significant factors based on the Chi-square test (p<0.05) were entered in multivariate logistic regression models which were used to examine the independent effects of independent factors (socio-demographic information, clinical features of TB, patient behaviors related to healthcare seeking, and patient access to TB health promotion) on delays. Potential confounders including age, gender, residence, district were adjusted for.

#### Qualitative analysis

Each interview was transcribed, carefully reviewed for accuracy. We analyzed key informant interviews and in-depth interviews separately using the framework approach [30]–[31]. The Framework approach to qualitative data analysis is a five-step process that involves: (i) *familiarization* (a process during which the researcher becomes immersed in the details of each transcript to gain a sense of the whole interview, prior to dividing them into sections and identifying recurrent themes), (ii) *developing a theoretical framework* (a process by which the researcher identifies recurrent and important themes in the transcripts), (iii) *indexing* (during which the researcher becomes further immersed in the data in order to refine identified themes and sub-themes), (iv) *summarizing data in an analytical framework* (during which the researcher reduces materials into understandable, but brief summaries of what was said by participants), and (v) *data synthesis, and interpretation* (which allows for comparison of themes and sub-themes against original transcripts, field notes, and audio recordings to ensure appropriate context) [30], [32]. Following the framework approach, as described above, transcripts of the interviews were coded and classified. We generated tabulations with category frequencies, category sequences and emerging themes. We then identified specific comments of respondents. These specific comments were then arranged into larger categories and themes [33]. The names of all participants in in-depth interviews and key informant interviews were removed from the quotations in the results to keep anonymity.

### Ethics Approval

The project proposal was approved by the Institutional Review Board of Third Military Medical University, Chongqing, China. Written informed consent was obtained from all participants once they agreed to take part in the study.

## Results

**Figure 2:**
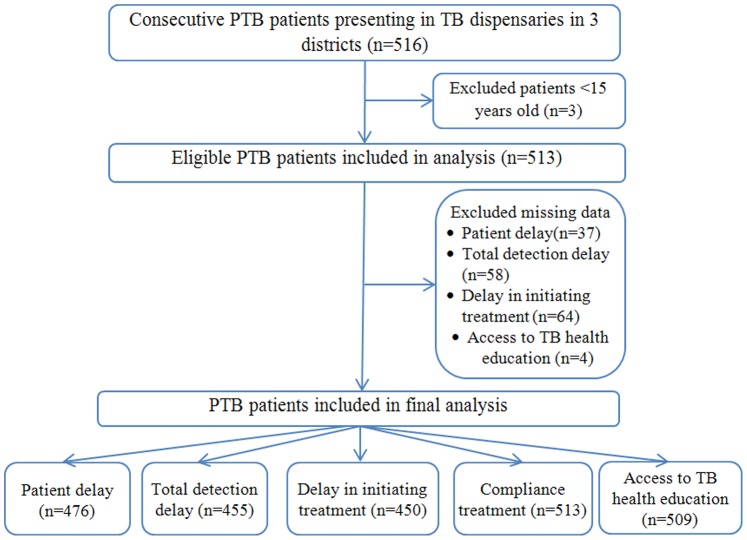
Flow diagram of participant inclusion in the study in Chongqing, China. This flow diagram showed the participants included in the study and final analysis of each related outcome.

Five hundred and sixteen patients (516) completed the questionnaire survey. Three participants aged <15 years old were excluded from the final analysis according to our inclusion criteria. After exclusion of missing data, information from 476 patients was used to analyze patient delay, 455 patients for total detection delay, and 450 for patients delay in initiating treatment. Data from 513 patients were entered onto the analysis of adherence to treatment, and 509 patients for access to health education (Figure 2).

### Demographic and Clinical Characteristics of Participants

Demographic and clinical characteristics of the respondent TB patients are presented in Table 1. A majority of participants were male patients aged 15 to 44 years. More than half were urban residents and had at least middle school education. About 24% (n = 114) had no health insurance, and 40.1% patients were below 2 poverty lines. A majority (n = 436; 97.5%) were newly diagnosed PTB patients. A high proportion of the patients (n = 337; 75.2%) were smear negative, and 94.1% (n = 448) had typical TB symptoms of chronic cough, haemoptysis, fever, night sweats, weakness and weight loss. Over ninety percent (n = 415; 91.2%) of the TB patients chose non-TB hospitals for their first visit at onset of symptoms and 76.5% (n = 399) consulted non-TB health facilities at least twice before their TB diagnosis.

**Table 1:** Demographic and clinical characteristics of the questionnaire respondents.^
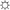
^

Characteristics	Frequency	Percent
**Age**(n = 476)		
16–45	335	70.4
46–60	100	21.0
>60	41	8.6
**Gender**(n = 475)		
Male	325	68.4
Female	150	31.6
**Ethnicity**(n = 474)		
Han Race	463	97.7
Others	11	2.3
**Marital status**(n = 473)		
Single	204	43.1
Married	234	49.5
Divorced	23	4.9
Windowed	12	2.5
**Residence**(n = 472)		
Rural	220	46.6
Urban	252	53.4
**Education**(n = 463)		
Primary and below	64	13.8
Middle school	266	57.5
College and above	133	28.7
**Health insurance**(n = 476)		
Yes	362	76.1
No	114	23.9
**District**(n = 476)		
JLP	178	37.4
SPB	198	41.6
YZ	100	21.0
**Personal income in past year**(n = 464)		
≤2PL	186	40.1
2–3	13	2.8
>3PL	265	57.1
**Type of patient**(n = 447)		
New	436	97.5
Retreatment	11	2.5
**AFB smear status** (n = 448)		
Negative	337	75.2
Positive	111	24.8
**Typical symptom**(n = 476)		
Yes	448	94.1
No	28	5.9
**First health facility for consultation**(n = 455)		
TB hospital	40	8.8
Non-TB hospital	415	91.2
**Number of Times health facility consulted before diagnosis**(n = 455)		
1	56	12.3
2	348	76.5
≥3	51	11.2
**Side effects when taking anti-TB drugs**(n = 510)		
Yes	150	29.6
No	360	70.4

Notes:

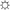
Missing data were excluded.

PL refers to local poverty line which is 3480 RMB Yuan per year since 2010.

TB refers to tuberculosis.

AFB smear status refers to Acid-Fast Bacilli (AFB) Smear status.

### Delay among PTB Patients

**Figure 3:**
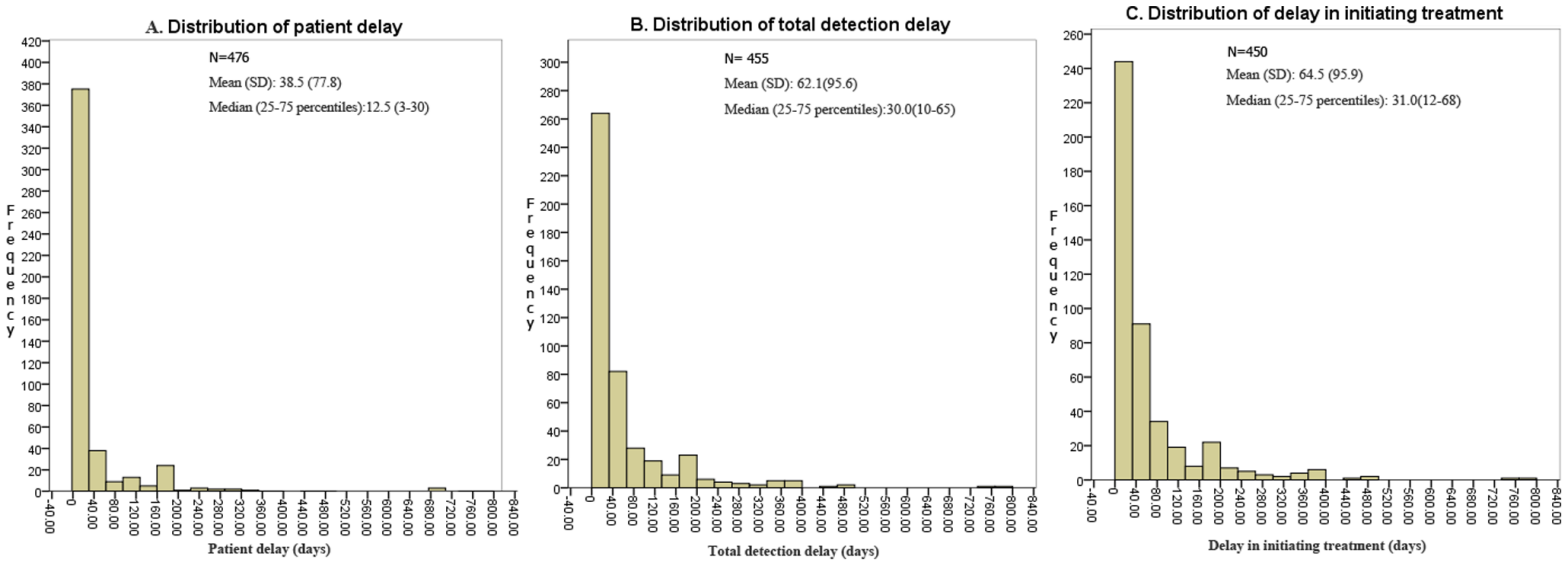
Lengths of different types of delay among TB patients in Chongqing, China. This figure showed lengths of patient delay (A), total detection delay (B) and delay in initiating treatment(C).

A median of 12.5 days (IQR 3–30 days) was observed for patient delay, with a mean of 38.5 days (SD 77.8) (Figure 3A). The total detection delay had a median of 30 days (IQR 10–65 days) and a mean of 62.1 days (SD 95.6) (Figure 3B). Delay in initiating treatment was a median of 31 days (IQR 12–68 days) and a mean of 64.5 days (SD 95.9) (Figure 3C).

Results of *X^2^* test indicated that widowed patients, urban residence, living in JLP district, personal income >3PL in the past year (about 1683$), negative AFB smear status, having typical TB symptoms of cough and sputum with blood, and low access to TB health education before infection with TB, were potentially associated with longer patient delay (>12.5 days from the appearance of symptoms to seeking healthcare) (*p*≤0.05). Similarly, risk factors for longer total detection delay (>30 days) included Han ethnicity, being divorced, living in JLP district, personal income >3PL in the past year (about 1683$), limited physical activity, negative AFB smear status, having typical TB symptom of cough, consulting a non-TB health facility ≥3 times before diagnosis, and lack of access to TB health education (*p*≤0.05). Risk factors for longer delay in initiating treatment (>31 days) included Han ethnicity, being divorced, living in JLP district, personal income >3PL in the past year (about 1683$), limited participation in physical activities, having negative AFB smear status, having typical symptoms of cough, consulting a health facility ≥3 times before diagnosis of TB, and lack of access to TB health education before TB diagnosis were (*p*≤0.05) (Table 2).

**Table 2:** Univariate analysis factors association with longer delay.^
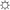
^

Categories	Delay≥12.5days(N = 238)	*P (X^2^test)*	Delay≥31days(N = 232)	*P (X^2^test)*	Delay≥31days(N = 232)	*P (X^2^test)*
**Age**						
16–45	157(46.9)	0.18	148(46.4)	0.17	149(47.8)	0.05
46–60	57(57.0)		59(61.5)		59(60.8)	
>60	22(53.7)		24(60.0)		24(58.5)	
**Gender**						
Male	156(48.0)	0.17	156(49.8)	0.51	157(50.5)	0.45
Female	82(54.7)		75(53.2)		75(45.3)	
**Ethnic**						
Han Race	232(50.1)	0.37	227(51.4)	0.03	228(52.2)	0.02
Others	4(36.4)		2(18.2)		2(18.2)	
**Marital status**						
Single	84(41.2)	<0.01	86(43.92)	0.03	87(45.1)	0.65
Married	131(56.0)		121(54.3)		122(55.0)	
Devoiced	14(60.9)		15(71.4)		14(70.0)	
Windowed	8(66.7)		7(58.3)		7(58.3)	
**Residence**						
Rural	97(44.1)	0.02	98(46.7)	0.15	98(47.3)	0.14
Urban	137(54.4)		129(53.5)		130(54.5)	
**Education**						
Primary and below	36(56.3)	0.25	37(60.7)	0.14	37(60.7)	0.15
Middle school	135(50.8)		129(51.2)		129(52.4)	
College and above	59(44.4)		58(45.3)		59(45.7)	
**Health insurance**						
Yes	175(48.3)	0.33	175(49.7)	0.41	175(50.6)	0.45
No	61(53.5)		56(54.4)		57(54.8)	
**District**						
SPB	65(32.8)	<0.01	113(71.5)	<0.001	113(73.4)	<0.01
JLP	124(69.7)		65(33.9)		65(33.9)	
YZQ	49(49.0)		53(50.5)		54(51.9)	
**Personal income in past year**						
<2PL	78(41.9)	0.02	73(41.2)	0.02	75(42.4)	<0.01
3-Feb	6(46.2)		5(38.5)		6(46.2)	
>3PL	146(55.1)		148(58.0)		146(58.4)	
**Physical activities**						
No	89(54.6)	0.13	94(59.9)	<0.01	94(60.3)	<0.01
Sometimes	110(48.5)		104(48.1)		105(49.3)	
Often	32(41.0)		29(38.7)		29(39.2)	
**Smoking**						
Non-smoking	146(49.8)	0.29	139(49.6)	0.71	139(50.9)	0.62
Smoking	60(46.5)		64(51.2)		64(50.4)	
Quit	32(59.3)		28(56.0)		29(58.0)	
**Alcohol drinking**						
Non-drinking	160(49.7)	0.97	157(50.9)	0.76	157(51.8)	0.79
Drinking	49(49.0)		49(51.6)		49(50.5)	
Quit	25(51.0)		22(47.8)		23(50.0)	
**Type of patient**						
New	224(51.4)	0.69	219(52.1)	0.89	220(53.5)	0.82
Retreatment	5(45.5)		5(50.0)		5(50.0)	
**AFB smear status**						
Negative	76(68.5)	<0.01	69(65.1)	<0.01	69(65.1)	<0.01
Positive	154(45.7)		155(47.7)		156(49.4)	
**Typical symptoms**						
Yes	231(51.6)	<0.01	225(52.4)	<0.01	226(53.4)	<0.01
No	5(17.9)		6(23.1)		6(22.2)	
**Cough**						
Yes	157(63.6)	<0.01	149(63.9)	<0.01	151(64.3)	<0.01
No	78(34.5)		81(37.0)		80(37.7)	
**Sputum with blood**						
Yes	54(41.9)	0.02	55(44.4)	0.88	55(44.7)	0.07
No	182(52.6)		176(53.3)		177(54.3)	
**First health facility for consultation**						
TB hospital	—	—	16(40.0)	0.15	16(41.0)	0.17
Non-TB hospital	—		215(51.8)		216(52.6)	
**Times to consult health facility before diagnosis**						
1	—	—	22(39.3)	<0.01	22(40.7)	<0.01
2	—		171(49.1)		172(50.0)	
≥3	—		38(74.5)		38(73.1)	
**Prescribing X-rays at first consultation**						
No	—	—	21(63.6)	0.13	21(67.7)	0.62
Yes	—		209(49.8)		210(50.4)	
**Prescribing sputum test at first consultation**						
No	—	—	163(48.8)	0.14	163(49.2)	0.085
Yes	—		68(56.7)		69(58.5)	
**Access to TB health education**						
Yes	62(40.3)	<0.01	59(39.1)	<0.01	59(40.4)	0.02
No	170(53.5)		168(56.0)		169(56.3)	
**TB knowledge**						
Yes	214(50.0)	0.77	206(50.2)	0.32	208(51.5)	0.36
No	22(47.8)		25(58.1)		24(58.5)	

Notes:

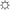
Missing data were excluded.

“—”refers this variable was not included in the logistic model for this independent variable.

PL refers to local poverty line which is 3480 RMB Yuan per year since 2010; TB refers to tuberculosis.

AFB smear status refers to Acid-Fast Bacilli (AFB) Smear status.

In the multivariate logistic regression analysis, factors that were significantly associated with patient delay ≥12.5 days were residence in JLP district [AOR (95% CI): 3.26(1.8, 5.9)], negative AFB smear status [AOR (95% CI): 1.8 (1.1, 3.0)], and having symptom of cough [3.3 (2.3, 4.8)]. Consulting a non-TB health facility ≥3 times before seeking care at a formal TB dispensary was a risk factor for both longer total detection delay [AOR (95% CI): 1.9(1.1, 3.3)] and longer delay in initiating treatment [AOR(95% CI): 1.88(1.1, 3.4)] (Table 3).

**Table 3:** Multivariate analysis for factors associated with delay.^
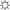
^

Variable	Delay≥12.5 days*AOR (95%CI)	Total detection delay Delay≥30 days§AOR (95%CI)	Delay in initiatingtreatment≥31 days†AOR(95%CI)
**Marital status**			
Single	Reference	Reference	:
Married	0. 5(0.1, 2.1)	1.2 (0.2, 9.6)	:
Devoiced	0.5 (0.1, 2.5)	0.9 (0.1, 7.4)	:
Windowed	0.6(0.1, 3.4)	1.1(0.7, 1.7)	:
**District**			
SPB	Reference	:	:
JLP	3.3(1.8, 5.9)	:	:
YZQ	0.7 (0.4, 1.3)	:	:
**Residence**			
Rural	Reference	:	:
Urban	1.5(0.9, 2.3)	:	:
**Income**			
≤2PL	:	Reference	Reference
2–3	:	0.4(0.8, 2.1)	0.7(0.1, 3.7)
>3PL	:	1.2 (0. 9, 1.6)	1.2(0.88, 1.6)
**Physical activities**			
No	:	Reference	Reference
Sometimes	:	0.4 (0. 9, 5.0)	0.1(0.1, 4.2)
often	:	0.67(0.5, 1.0)	0. 7(0.4,1.0)
**AFB smear status**			
Positive	Reference	Reference	Reference
Negative	1.8 (1.1, 3.0)	1.0 (0.5, 2.1)	0.9 (0.5, 1.9)
**Times to consult health facility before diagnosis**			
1	:	Reference	Reference
2	:	0.3(0.1, 1.0)	0.3(0.2, 1.1)
≥3	:	1. 9(1.1, 3.3)	1.9(1.1, 3.4)
**Access to TB health education**			
Yes	Reference	Reference	Reference
No	1.3(0.8, 2.1)	1.5 (0.8, 2.8)	1. 6(0.8, 2. 9)
Cough			
No	Reference	Reference	Reference
Yes	3.3 (2.3, 4.8)	1.7(0.9, 2.9)	0.9 (0.5, 1.6)
**Sputum with blood**			
Yes	Reference	:	:
No	1.3 (0.8, 2.1)	:	:

Note:

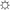
Missing data were excluded.

*Adjusted for age and gender.

§ Adjusted for age, gender, residence, district† Adjusted by age, gender, residence, district.

: refers this variable was not included in the logistic model for this independent variable.

PL refers to local poverty line which is 3480 RMB Yuan per year since 2010; TB refers to tuberculosis.

AFB smear status refers to Acid-Fast Bacilli (AFB) Smear status.

### Treatment and Management of TB Patients

**Figure 4:**
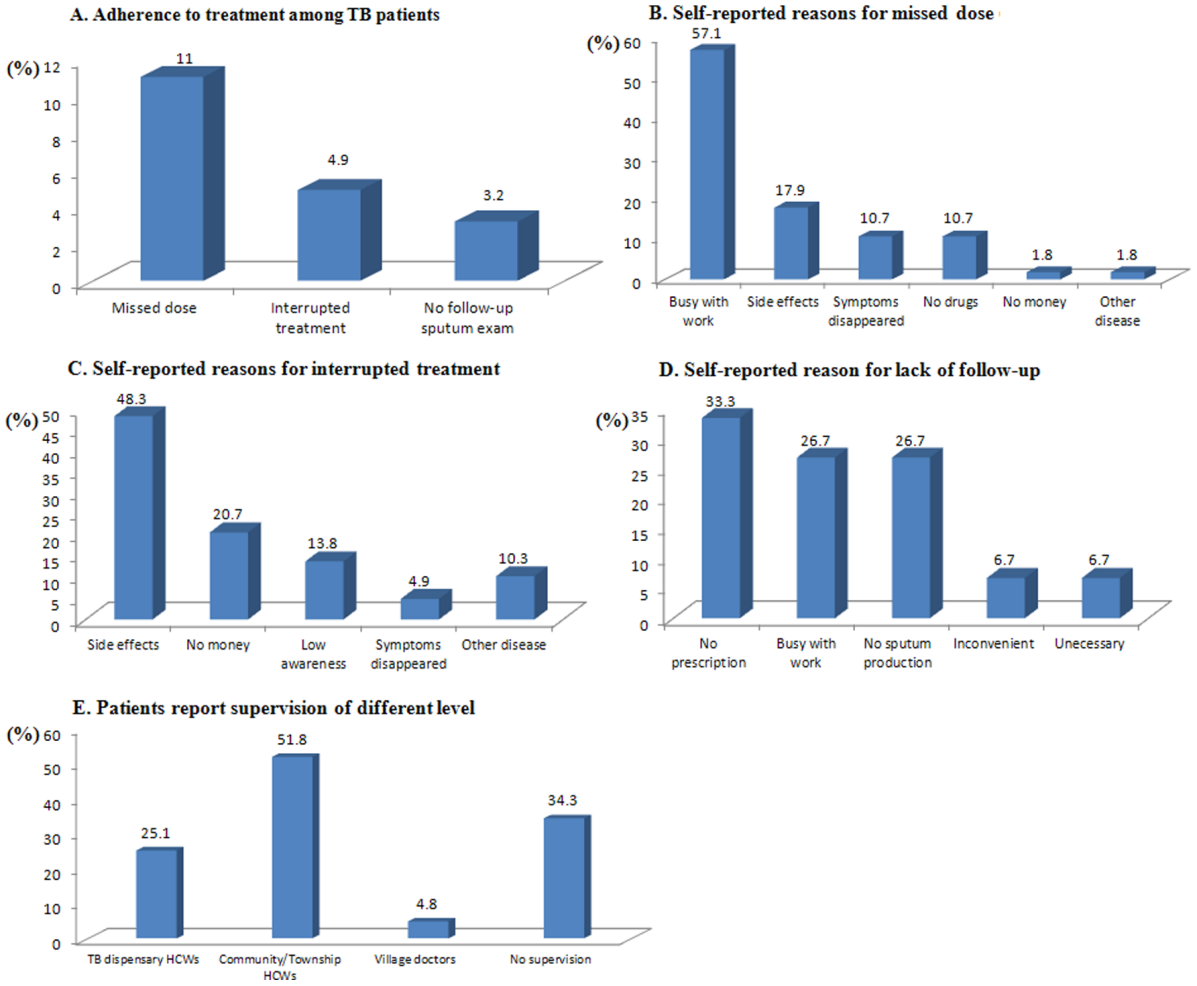
Adherence to treatment, self-reported reasons for non-adherence, and treatment supervision among TB patients in Chongqing, China. This figure presented the adherence to anti-TB treatment(A), self-reported reasons for missed dose (B), self-reported reasons for interrrupted treatment(C), self-reported reasons for lack of follow-up exam(D) and treatment supervision by HCWs of different levels(E).

The time interval between diagnosis and initiation of treatment ranged from 0 to 30 days (Mean = 2.0, SD = 3.2); 70.7% of patients (n = 363) took their anti-TB drugs within one day after diagnosis. Eleven percent of patients (n = 56) missed at least one dose, 4.9% (n = 25) had interrupted treatment, and 3.2% (n = 16) did not follow up with referral to sputum examination according to the standard treatment regimen (Figure 4A). Among patients missing doses of anti-TB drugs, many (n = 31; 57.1%) forgot to take their drugs because of work commitments, and 17.9% (n = 11) stopped taking drugs due to adverse effects of the drugs. Although 48.3% of patients experienced interrupted treatment due to adverse effects of anti-TB drugs, more than ^1^/_5_ of patients did so because of lack of money for treatment. More than ^1^/_3_ (33.3%) of patients who failed to follow-up the sputum exam reported no such recommendation by HCWs, and 26.7% did not adhere to referral to follow-up the sputum exam because of their busy work schedules (Figure 4B–C). As for DOT, 34.3% of patients (n = 176) were never monitored by HCWs, 25.1% of the patients (n = 129) were supervised by TB dispensary HCWs, 51.8% HCWs in community/township health centers and 4.8% by village doctors (Figure 4E). A majority of patients (n = 490; 95.5%) reported taking anti-TB drugs by themselves and only 1.2% of patients reported that HCWs supervised them while taking medications.

### Access to Health Education

**Figure 5:**
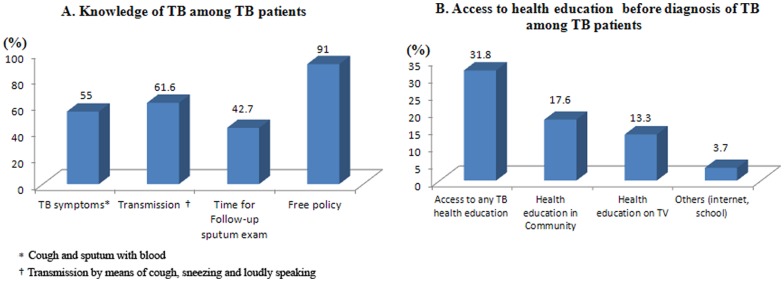
TB knowledge and access to TB health promotion among TB patients in Chongqing, China. This figure demonstrated current TB knowledge of TB patients (A) and access to TB health education before TB diagnosis (B).

Only 55% of patients (n = 280) knew about the common TB symptoms of cough and sputum with blood; 61.6% (n = 314) knew that transmission could occur through coughing, sneezing and speaking loudly, and 42.7% (n = 217) had knowledge of the timetable for a follow-up sputum exam (Figure 5A). With regard to TB patients’ access to TB health education before diagnosis with TB infection (Figure 5B); only 31.8% (n = 162) had accessed any type of TB health education. Of this number, 17.6% (n = 89) had access to TB information from the community, 13.3% (n = 68) from TV, and 3.7% (n = 19) from other sources (internet or school).

### Results of Qualitative Research

Findings of the qualitative research were consistent with results of the questionnaire survey. Analyses yielded 4 themes that characterized the perspectives of TB health providers and patients with regard to healthcare seeking behaviors related to TB care:

#### Theme 1: Health seeking behavior related to TB care

(a) Providers’ perspectives: TB patients had longer patient delay of 2–3 months on average. Some patients had delays of more than 1 year. A majority of the patients who experienced longer patient delays often consulted non-TB health facilities (e.g., private clinics, center of community health or general hospitals) for 2–3 times before receiving their diagnosis of TB. The providers felt that another importance cause of longer patient delay was patients’ poor TB knowledge and awareness, and treatment cough as cold or chronic respiratory disease in non-TB health facilities resulted in longer total detection delay. As one provider stated, *“On average, they patients come to us 2–3 months after onset of symptoms, some even 1–2 years. They thought cough was symptom of cold and didn’t bother about it. Usually they would visit the general hospital first where they would be treated for cold.”* A 38 year healthcare worker with several years of experience in TB control commented, “*They would not go to TB dispensary first because they have no awareness of TB; they think TB is a very horrible infectious disease they could not get. They usually buy anti-inflammatory drugs for their cough, or seek care in private clinics or the district hospital first”*. Another healthcare worker (aged 50 with many years of experience in TB control) added, “*it only after their symptoms do not improve in the non-TB hospitals that health provider in those facilities would order X-rays test.”*

(b) Patients’ perspectives: Half of the respondent TB patients mistook their TB signs and symptoms for cold and went to the general hospitals for treatment. A majority sought care in a TB facility after 0.5–3 months when their symptoms became severe. *“I felt tired at first, and I thought it was cold, and that it would go away. But I felt it became severe after about 2 months and I then went to our local general hospital where the doctor prescribed X-rays and asked me to come here (TB dispensary*)” (49 years old, unemployed TB patient).

#### Theme 2: Compliance with treatment

*(a)* Providers’ perspectives: The healthcare providers interviewed believed that about 90% TB patients compiled with TB treatment and follow-up appointments. There was a feeling that few interrupted their treatment because of busy work schedules, improvements in symptoms, concerns about side-effects, forgot to take their medication while travel or had financial difficulties. The comment that *“after treatment for two months, some patients give up treatment when their symptoms begin to disappear, when they experience side effects, were busy with work, or have no money”* was made by several of the health providers.

(b) Patients’ perspectives: All TB patients admitted that they complied with their treatment except one who noted that her treatment was interrupted for two months due to her busy work schedule. *“I adhere to my treatment mostly, but sometimes I forget it because of busy work, and I interrupted my treatment for two months because I went out of this city on business”* (47-year-old, self-employed TB patient).

#### Theme 3: Quality of Directly Observed Therapy (DOT)

(a) Providers’ perspectives: The HCWs interviewed noted that they usually monitor treatment by calling patients to remind them to take their medication and to keep their appointments. They noted that they usually would make repeated calls to patients who did not adhere to treatment according to clinic records. “*We call TB patients once a week or once a month and they would tell us about side-effect of treatment. We would ask them to go to the TB dispensary to take a test… we would educate them about the importance of compliance with treatment. But some patients would still interrupt their treatment, perhaps for economic reasons” to take drugs because of economic reason and side-effect.”* (59-year old TB control health worker). In another remark, a 50-year old TB control health worker noted, *“We talk to every new patients about the importance of adherence to treatment and* will give them a card to record when they take their drugs to prevent them from forgetting.”

(b) Patients’ perspectives: Less than half (6/15) of the patients agreed that TB control health workers monitored them by calling to remind them about taking their medications. However, some patients corroborated health workers’ claim about reaching out to TB patients to promote adherence. *“Doctors in community called me several times every month to remind me to take my medicines”* (Unemployed, 35 years old, female TB patient).

#### Theme 4: Access to TB health education

*(a)* Providers’ perspectives: Almost all of the TB health workers interviewed (9/10) commented that they provided TB education to their patients, giving them information about free treatment policy, and prevention transmission of TB to others, and the importance of informing their regular contacts to screen for TB. There was agreement that patients receive adequate information about the importance of adherence to treatment, management of treatment side-effect, and regular follow-up sputum test. “*We do tell patients that TB is a curable infectious disease, educate them on the mode of TB transmission, how to treat TB by taking anti-TB drugs as prescribed, the importance of follow-up tests, management of sputum to control infection, free treatment policy (free x-rays for two times, free sputum tests for 4 times), and control measures such as wearing respirators and ventilation of rooms.”* (50-year old TB control health provider).

*(*b) Patients’ perspectives*:* All patients mentioned education on free treatment policy; a majority (13/15) reported receiving education on prevention of TB transmission to others. About half reported receiving education on strengthening nutrition and regular follow-up sputum tests, management of side-effect of drugs, and the importance of reducing the consumption of alcohol. *“Doctor told me about free anti-TB drugs for 6 months, asked me to take life easy and emphasized the need to take my drugs every day. They told me that interruption of treatment would result in drug resistance, and that I cannot speak loudly without covering my mouth in the first 6 month of treatment. They also told me come for get drugs every month, take sputum test every month, take x-ray every 2–3 months.”* (34-year old TB patient).

## Discussion

When individuals experience signs and symptoms of TB, they should report promptly for diagnosis, and should, if found to have TB, commence treatment promptly, adhere to their treatment regimen, and remain in treatment until effectively cured. However, this process can be disrupted by multiple individual and health systems factors. For example, limited knowledge about signs and symptoms of TB, poor health seeking behavior, and poor management of the disease in health facilities result in delays in TB diagnosis and treatment, which in return, increase the risk of TB transmission and the potential for development of MDR-TB [34]. It is known that owing to limited awareness of their condition, only 47% of Chinese patients with TB symptoms seek healthcare in a timely fashion; only 59% comply with prescribed treatment, and less than 50% of general population have proper knowledge of the disease [21].

Findings from this study showed that ≥50% TB patients had longer patient delay, total detection delay, and delay in initiating treatment. Longer patient delay can be used to measure patient awareness of TB in the passive TB detection system. Longer total detection delay may comprehensively reflect the efficiency of TB case identification in TB control system. Longer delay in initiating treatment reflects barriers in access anti-TB treatment. Evidence from the literature indicates that these delays are associated with increased risk for development of MDR-TB. For example, one cross-sectional study in China found that delay in initiating TB treatment was associated with MDR-TB [14]. A case-control study in China found that MDR-TB was independently associated with onset of symptoms lasting >3 months before diagnosis [10]. Similarly, a cross-sectional study in Mexico observed that cough lasting more than 3 years was significantly associated with MDR-TB [11]. In addition, delays before treatment for active tuberculosis are likely to be associated with a greater number of secondary cases per index case which may explain the risk for development of MDR-TB posed by previous treatment [35]. Thus, TB patients with longer delays in this study have increased potential risk for developing MDR-TB [7]–[16].

Although the explanations for previous treatment as a risk factor for MDR-TB vary, poor treatment adherence, irregular treatment and poor quality of DOTS are often reported as significant factors. In Pakistan for example, prior history of incomplete treatment was independently associated with MDR [13]. The national survey in China revealed that more than 40% of patients with MDR tuberculosis had not completed their last course of treatment [33]. One meta-analysis found that MDR-TB in China was significantly associated with poor treatment adherence [9]. This study found that 2.5% patients were retreatment TB patients, 11% missed at least one dose and 4.9% had interrupted treatment. Therefore, those TB patients at higher risk of developing MDR-TB should be followed up, and urgent measures taken to facilitate completion of their treatment.

DOTS which was launched by WHO in 1992 [36], helps cure most TB cases and can help to prevent drug resistant TB [37]. Poor implementation of the DOT short-course program leads to monotherapy and intermittent treatment, which underpin the emergence of TB drug resistance [37]. A study in Taiwan found significant negative correlation between the coverage rates of DOTS and DOTS-Plus programs and the rates of acquired MDR-TB [18]. Similarly, a meta-analysis found that MDR-TB in China was significantly associated with poor quality DOTS [9]. In China, DOTS was initiated in 1992 and DOTS coverage was reported at 100% [38]. WHO has called China’s National Tuberculosis Control Program (NTP) with the DOTS strategy ‘one of the most successful DOTS-programs in the world’ [39]. However, there is still a gap between national policies and actual practices. This study observed that more than ^1^/_3_ of TB patients were never monitored by any HCWs and 95.5% TB patients took anti-TB drugs by themselves. Therefore those TB patients who did not receive DOT were at risk for development of MDR-TB. Strengthening follow-up of patients with tuberculosis is one important opportunity for improvement of adherence to TB treatment.

Previous systematic reviews reported that patient and diagnostic delays in TB care are mediated by individual and health facility factors [34]. This study further demonstrates that poor TB knowledge/awareness, longer interval between onset of TB symptoms and healthcare seeking in designated TB facilities, and consulting a non-TB health facility were associated with diagnostic and treatment delays. These factors can be addressed by effective TB health promotion which includes individual education and empowerment, community empowerment, health systems strengthening, interagency partnerships, and intersectional collaboration [40]–[42]. TB health education/health promotion in China was emphasized by the *Guideline on Enforcement of Chinese Tuberculosis Control Program* in 2008 [43], with a targeted of raising population core knowledge of TB among the public to83% by 2010. However, this study shows that that only 31.8% TB patients ever accessed TB health education before diagnosis with TB, and less than 70% of the TB patients had TB knowledge. These results highlight the need to explore measures to improve the effectiveness of current TB health education.

Our qualitative results indicate that seeking care at non-TB health facilities was associated with diagnostic and treatment delays. Owing to lack of training in TB care, these facilities typically engage in activities that do not facilitate prompt TB care, including for example, treating TB-related cough as common cold, and not referring patients promptly to TB dispensaries. With regard to the barriers caused by patients socioeconomic, it is important that to develop policies that remove patients’ financial impediments in access to TB care [34], though for example, the expansion of the existing national TB free treatment policy to cover the cost of more visits, tests and treatments. Similarly, integration of all sectors of the healthcare system within the overall national TB control program in urban and rural settings is important as this may help to reduce some of the barriers against effective and prompt referrals [42]. Capacity building including training of HCWs, particularly in the provision of community-based TB education, case detection, and referral are important public health actions to deal with delays and reduce the development of MDR-TB.

In addition, social determinants of health (SDH) associated with TB control are well recognized. TB is a disease of poverty and its control requires the integration of social, economic, and environmental approaches with medical interventions [44]. This study confirmed that patients’ vulnerable socio-economic position (long working hours and low income) increased the risk for patient delay and treatment non-adherence. Given the relationship between social determinants and MDR-TB, actions to address the social determinants of TB are important for TB control and reduction in the prevalence of MDR-TB [39], [45]–[46].

Although this study has identified numerous potential challenges facing MDR-TB prevention throughout the process of seeking care, diagnosis, and treatment by TB patients, an important limitation relates to the fact that the study did not explore the outcome of treatment for patients who experienced delays and non-adherence to treatment. The study therefore could not analyze the association between delay, treatment non-adherence and the development of MDR-TB.

In conclusion, improving general TB control is important for the prevention of MDR-TB in countries with high TB burden. Based on results of this study, particular attention must be given to early case detection, prompt initiation of treatment and improvement in the quality of DOT in order to prevent MDR-TB from previous TB treatment. Therefore, strategies focused on improvement in the capacity of HCWs to effectively diagnose TB and manage TB are urgently needed. Efforts should also be made to improve DOT quality by strengthening training of health workers. It is equally important to conduct implementation studies to identify strategies to improve the effectiveness of TB health promotion. It is imperative to take measures to addressing the financial and health system issues in Chinese TB control [34] in order to remove barriers in access to TB care.
